# Development and Validation of an m6A RNA Methylation Regulator-Based Signature for Prognostic Prediction in Cervical Squamous Cell Carcinoma

**DOI:** 10.3389/fonc.2020.01444

**Published:** 2020-08-21

**Authors:** Jingxin Pan, Lichao Xu, Hongda Pan

**Affiliations:** ^1^Department of Internal Medicine, The Second Affiliated Hospital of Fujian Medical University, Quanzhou, China; ^2^Department of Interventional Radiology, Fudan University Shanghai Cancer Center, Shanghai, China; ^3^Department of Oncology, Shanghai Medical College, Fudan University, Shanghai, China

**Keywords:** m6A, RNA methylation, cervical squamous cell carcinoma, prognosis, experimental validation

## Abstract

**Background:** Cervical squamous cell carcinoma (CESC) is one of the most common causes of cancer-related death worldwide. N6-methyladenosine (m6A) plays an important role in various cellular responses by regulating mRNA biology. This study aimed to develop and validate an m6A RNA methylation regulator-based signature for prognostic prediction in CESC.

**Methods:** Clinical and survival data as well as RNA sequencing data of 13 m6A RNA methylation regulators were obtained from The Cancer Genome Atlas (TCGA) CESC database. Consensus clustering was performed to identify different CESC clusters based on the differential expression of the regulators. LASSO Cox regression analysis was used to generate a prognostic signature based on m6A RNA methylation regulator expression. The effect of the signature was further explored by univariate and multivariate Cox analyses.

**Results:** Four regulators (RBM15, METTL3, FTO, and YTHDF2) were identified to be aberrantly expressed in CESC tissues. A prognostic signature that includes ZC3H13, YTHDC1, and YTHDF1 was developed, which can act as an independent prognostic indicator. Significant differences of survival rate and clinicopathological features were found between the high- and low-risk groups. The results of bioinformatics analysis were then validated in the clinical CESC cohort by qRT-PCR and immunohistochemistry staining.

**Conclusion:** In the present study, we developed and validated an m6A RNA methylation regulator-based prognostic signature, which might provide useful insights regarding the development and prognosis of CESC.

## Introduction

Cervical squamous cell carcinoma (CESC) is the fourth most commonly diagnosed cancer and the fourth leading cause of cancer-associated mortality in women worldwide (1). Persistent infection with human papillomavirus (HPV) is the predominant cause of CESC (2). The development of accurate prognostic predictors in order to establish personalized treatment for CESC patients is crucial.

N6-methyladenosine (m6A) modification is one of the most prevalent modification in mRNA in eukaryotic cells. m6A RNA modification plays crucial roles in many processes of gene regulation such as mRNA stability, splicing, and translation (3). m6A RNA modification can be installed enzymatically by various methyltransferases, termed m6A “writers” (METTL3, METTL14, WTAP, KIAA1429, RBM15, and ZC3H13). m6A in RNA can be removed by demethylases, termed m6A “erasers” (FTO and ALKBH5). Proteins that selectively bind m6A can be defined as m6A “readers” (HNRNPC, YTHDF1, YTHDF2, YTHDC2, and YTHDC1) that exert regulatory functions by selective recognition of methylated RNA (4). Emerging evidence has revealed the cancer promoter or suppressor role of m6A regulators in the development of various malignancies (5–7), whereas the correlation between prognosis of CESC and m6A RNA methylation regulators is still unclear.

**Figure 1:**
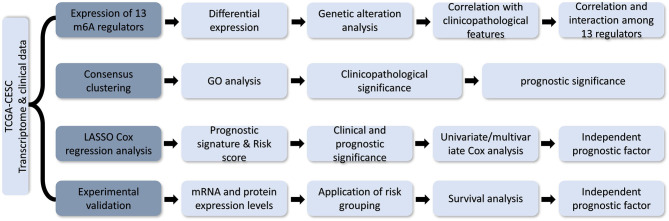
Flow chart of development and validation of an m6A RNA methylation regulator-based prognostic signature for CESC.

In this study, the differential expression of m6A RNA methylation regulators was analyzed using the RNA sequencing data from the TCGA-CESC dataset. The interactions among these regulators and their correlation with clinicopathological features were evaluated. Consensus clustering was used to identify two clusters of CESC patients to predict clinical outcome. By LASSO Cox analysis, a three-gene prognostic signature was generated. Moreover, the bioinformatics prediction was experimentally validated in a clinical CESC cohort (Figure 1). The m6A RNA methylation regulator-based prognostic signature can act as a useful tool for predicting the survival outcomes of CESC patients.

## Materials and Methods

### TCGA Data Acquisition

RNA transcriptome data in the Fragments Per Kilobase per Million (FPKM) format and the clinical and survival data of CESC patients were downloaded from TCGA database (https://cancergenome.nih.gov/). All analyses were performed according to the publication guidelines of TCGA. After duplicate samples from the same patients were excluded, a total of 304 CESC samples and three normal tissue samples were enrolled for subsequent analysis. Thirteen well-acknowledged m6A RNA methylation regulators (YTHDC1, YTHDC2, YTHDF1, YTHDF2, ALKBH5, FTO, METTL3, METTL14, HNRNPC, WTAP, RBM15, KIAA1429, and ZC3H13) were selected for further analysis according to previously published literature (8).

### Bioinformatics Analysis

Differential expressions of 13 m6A methylation regulators between different sample groups were identified by “limma” package in R software. Gene expression levels, as well as their correlation with clinicopathological features, were visualized by heatmaps generated with “pheatmap” package. The “corrplot” package was employed to reveal the correlation among m6A RNA methylation regulators. Interactions among m6A RNA methylation regulators were analyzed and a protein–protein interaction network was established and visualized by the STRING and Cytoscape 3.6.0. The genetic alterations of the m6A methylation regulators were analyzed by cBioPortal using data from TCGA. The CESC cohort was clustered into different groups by consensus expression of m6A RNA methylation regulators with “ConsensusClusterPlus” package. A “survminer” package in R software was used to determine the best cutoff of the expression value for survival analysis. Gene ontology (GO) annotation were performed by “clusterProfiler” package and visualized using circos plots generated by the “ggplot2” package.

### Construction of the Prognostic Signature

All m6A methylation regulators were included in the Least Absolute Shrinkage and Selection Operator (LASSO) Cox regression model to construct the powerful prognostic signature and calculate a coefficient for each gene. A risk score for each patient was calculated as the sum of each gene's score, which was obtained by multiplying the expression of each gene and its coefficient. The sensitivity and specificity of the prognostic signature were accessed by receiver operating characteristic (ROC) curves and area under the ROC curves (AUC values).

### Experimental Validation

One hundred twenty CESC tissues and paired normal tissues were obtained from Outdo Biotech (Shanghai, China). The mRNA and protein expression of ZC3H13, YTHDC1, and YTHDF1 were quantified by immunohistochemistry (IHC) staining and quantitative real-time PCR (qRT-PCR), as per previously described methods (9, 10). GAPDH was used as internal standard for normalization in qRT-PCR. Primer sequences of genes measured in this study were listed in Table S1. The validation cohort was grouped into low- and high-risk groups according to the risk scores calculated by the TCGA cohort. Written informed consent was obtained from all the patients. The validation study was approved by the Ethics Committee of Fudan University.

### Statistical Analysis

The chi-square test was used to compare the clinicopathological features between different groups. The Student's *t*-test (two-tailed) was applied to compare the differences between groups. Univariate and multivariate Cox regression analyses were used to identify the independent prognostic factors for patients with CESC. Kaplan–Meier method and log-rank test were used to compare the overall survival (OS) difference between different groups. Data analysis was performed with either GraphPad Prism 7.0 (GraphPad, San Diego, CA, USA) or SPSS v23.0 (IBM Corp., Armonk, NY, USA). All statistical tests were two-sided. A *P* < 0.05 was considered statistically significant.

## Results

### Expression Profile of m6A RNA Methylation Regulators in CESC

**Figure 2:**
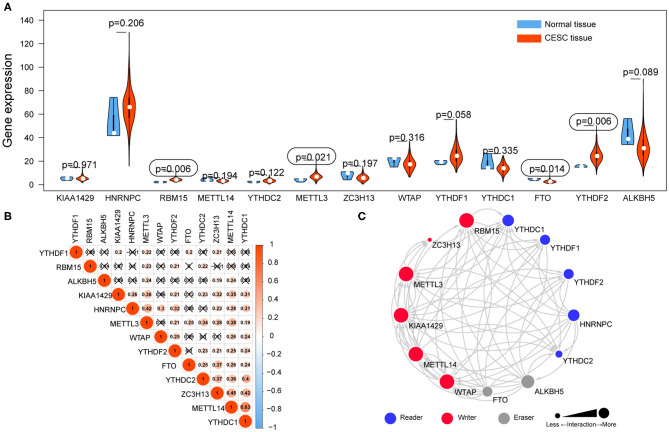
The expression of 13 m6A RNA methylation regulators in TCGA-CESC cohort. **(A)** The violin plot showed the significantly differentially expressed m6A RNA methylation regulators between CESC tissues and the normal tissues. **(B)** The correlations among m6A RNA methylation regulators were analyzed by Pearson correlation. **(C)** PPI network showed the interactions among m6A RNA methylation regulators.

The mRNA expression levels of m6A RNA methylation regulators were analyzed using transcriptome data in FPKM format. The differential expression of 13 regulators between CESC and normal tissues was demonstrated by a violin plot (Figure 2A). The mRNA expression levels of three regulators (RBM15, METTL3, and YTHDF2) were significantly increased, and FTO was decreased in CESC compared with normal tissues. No significant difference was found for the other nine regulators.

### Correlation and Interaction Among m6A RNA Methylation Regulators in CESC

Correlations among the mRNA expression levels of 13 m6A RNA methylation regulators were analyzed by Pearson correlation analysis (Figure 2B), and the protein–protein interactions (PPIs) were retrieved via String database (Figure 2C). The results showed that all the regulators were positively correlated with each other. Notably, YTHDC1 was significantly correlated with METTL14 (*r* = 0.63). The PPI network revealed that five writers (METTL3, METTL14, RBM15, KIAA1429, and WTAP) were all significantly correlated with each other, as well as readers and erasers. Interactions were founded to be few among the two erasers and five readers in the PPI network.

### Genetic Alteration of m6A RNA Methylation Regulators in CESC

**Figure 3:**
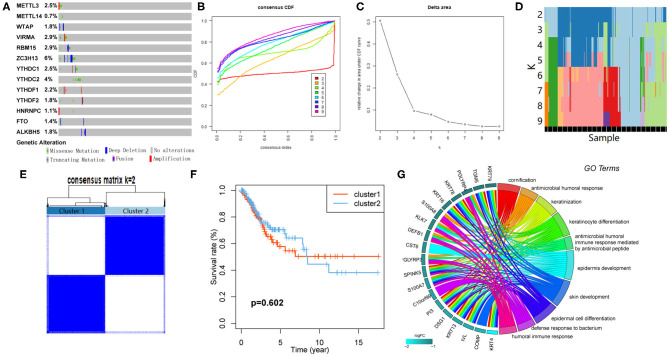
Consensus clustering based on the expression of m6A RNA methylation regulators. **(A)** Genetic alteration was analyzed *via* cBioPortal database. **(B)** Area under cumulative distribution function (CDF) curve when index *k* ranges from 2 to 10. **(C)** Changes of length and slope of CDF curve when index *k* ranges from 2 to 10. **(D)** Distribution of each sample in different clusters when *k* ranges from 2 to 10. **(E)** The overlap between clusters when *k* = 2. **(F)** The Kaplan–Meier survival analysis for Clusters 1 and 2. **(G)** GO analysis for the DEGs between Clusters 1 and 2.

The CNV and mutation of m6A RNA methylation regulators were analyzed via the cBioPortal database using TCGA data to investigate the effects of genetic alteration on the gene expression (Figure 3A). The results revealed that the frequencies of genetic alteration for ZC3H13 were 6%, and the most frequent alteration was deep deletion. Frequencies for other regulators were <3%, indicating that changes in the expression levels of these regulators were not caused by genetic alteration.

### Consensus Clustering Identified Two Clusters of Patients With CESC

CESC cohort could be divided into several clusters according to the consensus of mRNA expression of the 13 m6A RNA methylation regulators. When the clustering index “*k*” increased from 2 to 9, *k* = 2 was demonstrated to be the optimal point to obtain the largest differences between clusters (Figures 3B,C). Besides, the interference between clusters was minimal when *k* = 2 (Figures 3D,E). Subsequently, the CESC cohort was divided into two clusters, namely, Cluster 1 and Cluster 2 (Figure 3E). However, no survival difference between the two clusters was found by Kaplan–Meier survival analysis (Figure 3F).

### GO Analysis for Differentially Expressed Genes (DEGs) Between Clusters

One hundred ten DEGs between clusters were identified to investigate the differences of biological roles between these clusters. GO analyses for biological processes were conducted and showed that DEGs were mainly enriched in biological processes associated with the development of the immune system (Figure 3G).

### Clinicopathological Differences Between the Clusters

**Figure 4:**
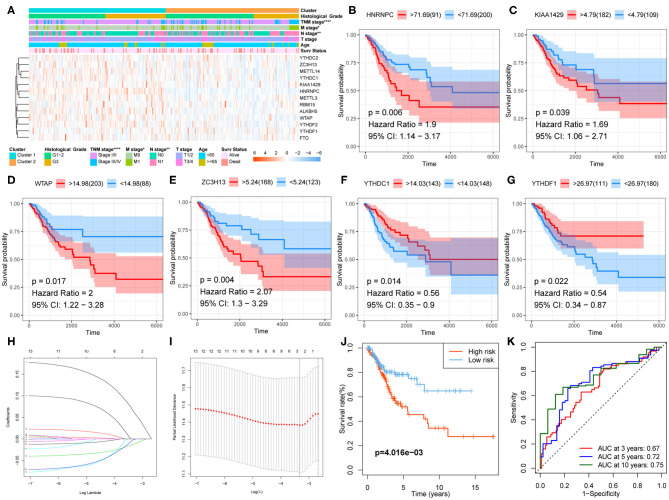
Clinical significance of clustering and construction of the prognostic signature. **(A)** The clinicopathological differences between the two clusters. **(B–G)** Kaplan–Meier survival curve for HNRNPC, KIAA1429, WTAP, ZC3H13, YTHDC1, and YTHDF1. **(H,I)** The prognostic signature constructed by the minimum criterion of LASSO Cox regression algorithm. **(J)** The Kaplan–Meier survival analysis for high- and low-risk groups. **(K)** ROC curve was used to evaluate the prediction efficiency of the prognostic signature.

Correlation between the clustering and clinicopathological characteristics was then analyzed between the two clusters. As shown in Figure 4A, Cluster 1 was significantly associated with advanced N stage, M stage, and TNM stage.

### Development of a Prognostic Signature

Kaplan–Meier survival analysis was conducted for these 13 regulators to explore the prognostic significance of the m6A RNA methylation regulators in CESC. The results showed that high expression levels of HNRNPC, KIAA1429, WTAP (Figures 4B–E), and ZC3H13 were correlated with poor survival, whereas high expression levels of YTHDC1 and YTHDF1 were associated with longer OS (Figures 4F,G).

A prognostic signature, including ZC3H13, YTHDC1, and YTHDF1, was developed using the LASSO Cox regression model according to the minimum criterion (Figures 4H,I). The coefficients of ZC3H13, YTHDC1, and YTHDF1 were 0.0644, −0.0016, and −0.012, respectively. The risk score for each CESC patient was therefore calculated with the following formula: Risk Score = 0.0644 ^*^ ZC3H13 – 0.0016 ^*^ YTHDC1 – 0.012 ^*^ YTHDF1. Then, the CESC cohort was divided into low- and high-risk groups on the basis of the median risk score.

### Prognostic and Clinicopathological Differences Between Low- and High-Risk Groups

**Figure 5:**
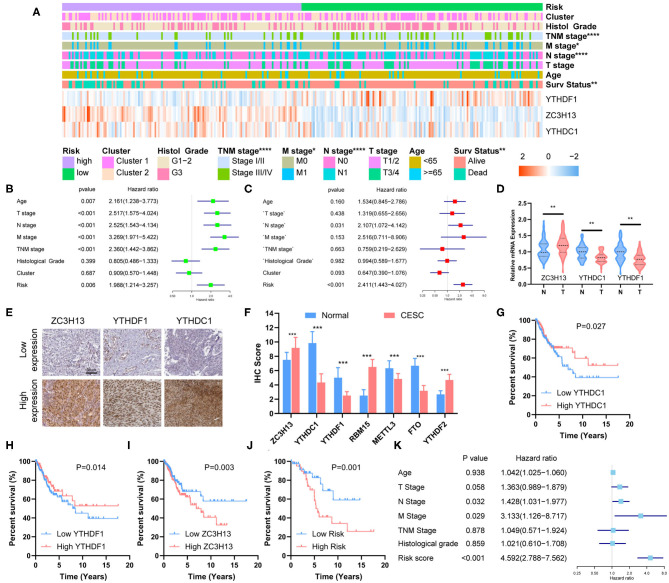
Clinical significance of risk grouping and experimental validation. **(A)** The clinicopathological differences between the high- and low-risk groups. **(B)** Univariate Cox analysis of the clinicopathological features and risk score. **(C)** Multivariate Cox analysis identified the independent prognostic predictors. **(D)** mRNA levels of ZC3H13, YTHDC1, and YTHDF1 in CESC and normal tissues were measured by qRT-PCR. **(E)** Representative IHC staining for ZC3H13, YTHDC1, and YTHDF1 in CESC and normal tissues (scale bar: 50 μm). **(F)** Protein expression of RBM15, METTL3, FTO, YTHDF2, ZC3H13, YTHDC1, and YTHDF1 was measured by IHC. **(G–I)** Kaplan–Meier survival analysis for patients with high or low expression levels of YTHDC1, YTHDF1, and ZC3H13. **(J)** Kaplan–Meier survival analysis for patients in low- or high-risk groups. **(K)** Multivariate COX analysis identified the independent prognostic predictors in the clinical CESC cohort.

Kaplan–Meier survival analysis was conducted to validate the prognostic value of risk grouping. The results revealed that the high-risk group had a worse overall survival than the low-risk group (*P* = 4.016e−03) (Figure 4J). Time-dependent ROC curve was used to assess the specificity and sensitivity of the prognostic signature. The area under the curve (AUC) at 3, 5, and 10 years was 0.67, 0.72, and 0.75, respectively, suggesting good prediction performances (Figure 4K). The high-risk group was significantly associated with advanced N stage (*P* < 0.05), M stage (*P* < 0.0001), TNM stage (*P* < 0.0001), and poor survival (*P* < 0.01) (Figure 5A).

### The Prognostic Signature Acts as an Independent Prognostic Predictor

Univariate and multivariate Cox analyses were performed to identify the independent prognostic predictors for CESC patients. The univariate Cox analysis showed that the Age, T stage, N stage, M stage, TNM stage, and Risk Score were significantly associated with the survival (Figure 5B). The multivariate Cox regression model showed that only Risk Score (*P* < 0.001, HR = 2.411, 95% CI = 1.443–4.027) and N stage (*P* = 0.031, HR = 2.107, 95% CI = 1.072–4.142) were the independent prognostic factors for CESC (Figure 5C).

### Experimental Validation

The mRNA expression of ZC3H13, YTHDC1, and YTHDF1 was measured with qRT-PCR, and the results showed that ZC3H13 was significantly upregulated in CESC tissues, whereas YTHDC1 and YTHDF1 were significantly downregulated in CESC tissues (Figure 5D). The differential expressions were also confirmed by IHC staining (Figures 5E,F). Low YTHDC1 and YTHDF1 expression was associated with poor survival (Figures 5G,H), and high level of ZC3H13 was correlated with lower survival rate (Figure 5I). Risk score was calculated for each patient in the validation cohort according to the formula and coefficient obtained from the TCGA cohort. Fifty-seven patients were identified as a high-risk group, and the rest of the 63 patients were categorized into a low-risk group. The survival rate was significantly lower in the high-risk group in comparison with that in the low-risk group (Figure 5J). Multivariate Cox analysis showed that the risk score, along with the N stage and M stage, was an independent prognostic factor for the overall survival of CESC patients in the validation cohort (Figure 5K). The prognostic significance of the three-gene signature in the validation cohort was in accordance with that of TCGA cohort.

## Discussion

Globally, CESC is one of the most common types of cancer and exists as a major therapeutic challenge (8, 11). One major cause of high mortality of CESC is high levels of patient relapse and mortality after treatment. The carcinogenesis of CESC is a complex multistep process characterized by a broad spectrum of molecular abnormalities that offers numerous potential therapeutic targets (12). Understanding the mechanisms of action of these molecules is crucial for their potential therapeutic use. Human papillomavirus (HPV) infection plays an important role in cervical cancer (13). m6A is the most abundant internal modification of RNA in eukaryotic cells (14). Emerging evidence suggests that aberrant m6A RNA methylation plays a critical role in cancer through various mechanisms (15, 16).

The level of m6A methylation is regulated by methyltransferases (writers), demethylases (erasers), and binding proteins (readers). Previous studies have demonstrated that m6A RNA methylation regulators were aberrantly expressed in various types of cancers and exert roles of promoter or suppressor of cancers (17). Zhang et al. demonstrated m6A regulator-mediated methylation modification patterns and tumor microenvironment infiltration characterization in gastric cancer (5). METTL3 is significantly upregulated in hepatoblastoma, and it regulates β-catenin to promote tumor proliferation (17). Yang et al. revealed that FTO promoted melanoma tumorigenesis and anti-PD-1 resistance and suggest that the combination of FTO inhibition with anti-PD-1 blockade may reduce the resistance to immunotherapy in melanoma (18). However, the roles of m6A methylation regulators in CESC are unclear.

In the present study, a three-gene prognostic signature, consisting of ZC3H13, YTHDC1, and YTHDF1, was developed and demonstrated good performance for predicting the survival outcome of CESC. Additionally, we validated the results of bioinformatics analysis with a clinical CESC cohort. The protein and mRNA expression of ZC3H13, YTHDC1, and YTHDF1 were measured by IHC and qRT-PCR. The results of experimental validation are consistent with those of bioinformatics prediction, suggesting that the prognostic signature might serve as a useful tool for predicting survival outcomes of CESC.

ZC3H13 is a canonical CCCH zinc finger protein and plays an important role in modulating RNA m6A methylation in the nucleus (19). Zhu et al. reported that ZC3H13 might be an upstream regulator of Ras-ERK signaling pathway and suppressed invasion and proliferation of colorectal cancer (20). Xiao et al. reported that the nuclear m6A reader protein YTHDC1 impacts mRNA splicing, providing a transcriptome-wide glance of splicing changes affected by this mRNA methylation reader protein (21, 22). YTHDF1 is a core factor in RNA methylation modification. Bai et al. demonstrated that knocking down the expression of YTHDF1 significantly inhibited the colorectal cell progression, and silencing YTHDF1 significantly inhibited Wnt/β-catenin pathway activity in colorectal cells (23). Han et al. reported that loss of YTHDF1 in classical dendritic cells enhanced the cross-presentation of tumor antigens and the cross-priming of CD8+ T cells *in vivo*. The therapeutic efficacy of PD-L1 checkpoint blockade is enhanced in Ythdf1^−/−^ mice, implicating YTHDF1 as a potential therapeutic target in anticancer immunotherapy (24).

In conclusion, our study revealed that the aberrant expression of m6A RNA methylation regulators is significantly correlated with the survival and clinicopathological characteristics of patients with CESC. The m6A RNA methylation regulator-based prognostic signature can effectively predict the prognosis of CESC patients.

## Data Availability Statement

Publicly available datasets were analyzed in this study, these can be found in The Cancer Genome Atlas (https://portal.gdc.cancer.gov/).

## Ethics Statement

The studies involving human participants were reviewed and approved by the Ethics Committee of Fudan University. The patients/participants provided their written informed consent to participate in this study.

## Author Contributions

HP, LX, and JP conceived and designed the experiments. JP analyzed the data and drafted the manuscript. LX discussed, contributed to the data analysis, and contributed to the sampling. All authors read and approved the final manuscript.

## Conflict of Interest

The authors declare that the research was conducted in the absence of any commercial or financial relationships that could be construed as a potential conflict of interest.
