# Supplementary material for: Development and Validation of an m6A RNA Methylation Regulator-Based Signature for Prognostic Prediction in Cervical Squamous Cell Carcinoma
Source: Front Oncol. 2020 Aug 21;10:1444. doi: 10.3389/fonc.2020.01444 (PMC7472601; doi:10.3389/fonc.2020.01444)
Supplement: Supplementary file 1 [file Table_1.docx]

| **Table S1. Primer sequences used in qRT-PCR** | | |
| --- | --- | --- |
| ZC3H13 | Forward | GTGCCGTAACTGGCTGAAGA |
|  | Reverse | CCTTTACCACGAGGTGAAGGG |
| YTHDC1 | Forward | CTTCTGATGAGCAAGGGAACAA |
|  | Reverse | GGCCTCACTTCGAGTGTCATAA |
| YTHDF1 | Forward | ATACCTCACCACCTACGGACA |
|  | Reverse | GTGCTGATAGATGTTGTTCCCC |
| GAPDH | Forward | TGACTTCAACAGCGACACCCA |
|  | Reverse | CACCCTGTTGCTGTAGCCAAA |
